# A novel DNA damage repair-related gene signature predicting survival, immune infiltration and drug sensitivity in cervical cancer based on single cell sequencing

**DOI:** 10.3389/fimmu.2023.1198391

**Published:** 2023-06-28

**Authors:** Xiaoqing Xiang, Jiawen Kang, Jingwen Jiang, Yaning Zhang, Yong Zhang, Lesai Li, Xiaoning Peng

**Affiliations:** ^1^Department of Internal Medicine, Medical College of Hunan Normal University, Changsha, Hunan, China; ^2^The High School Attached to Hunan Normal University, Changsha, China; ^3^Department of Gynecologic Oncology, Hunan Cancer Hospital/the Affiliated Cancer Hospital of Xiangya School of Medicine, Central South University, Changsha, Hunan, China

**Keywords:** cervical cancer, DNA damage repair, prognostic signature, bulk sequencing, single-cell RNA sequencing analysis, immune infiltration, drug sensitivity

## Abstract

**Background:**

Aberrant DNA damage repair (DDR) is one of the hallmarks of tumors, and therapeutic approaches targeting this feature are gaining increasing attention. This study aims to develop a signature of DDR-related genes to evaluate the prognosis of cervical cancer (CC).

**Methods:**

Differentially expressed genes were identified between high and low DDR groups of cells from the single-cell RNA sequencing dataset GSE168652 based on DDR scores. Using the ssGSEA and WGCNA methods, DDR-related differentially expressed genes were identified from different patients within the TCGA-CESC cohort. Using Cox analysis and LASSO regression analysis, a DDR-related gene signature was constructed based on the intersection of two groups of differentially expressed genes and DDR-related genes from WGCNA, and validated in GSE52903. Immune cell infiltration analysis, mutation analysis, survival analysis, drug sensitivity analysis, etc., were performed in different groups which were established based on the DDR gene signature scoring. A key gene affecting prognosis was selected and validated through biological experiments such as wound healing, migration, invasion, and comet assays.

**Results:**

A novel DDR-related signature was constructed and the nomogram results showed this signature performed better in predicting prognosis than other clinical features for CC. The high DDR group exhibited poorer prognosis, weaker immune cell infiltration in the immune microenvironment, lower expression of immune checkpoint-related genes, lower gene mutation frequencies and more sensitivity to drugs such as BI.2536, Bleomycin and etc. *ITGB1*, *ZC3H13*, and *TOMM20* were expressed at higher levels in CaSki and HeLa cells compared to ECT1 cells. Compared with the native CaSki and HeLa cells, the proliferation, migration, invasion and DDR capabilities of CaSki and HeLa cell lines with *ITGB1* suppressed expression were significantly decreased.

**Conclusion:**

The 7 DDR-related gene signature was an independent and powerful prognostic biomarker that might effectively evaluate the prognosis of CC and provide supplementary information for a more personalized evaluation and precision therapy. *ITGB1* was a potential candidate gene that may affect the DDR capacity of CC cells, and its mechanism of action was worth further in-depth study.

## 1 Introduction

According to the 2021 Global Cancer Statistics Report, CC ranks as the fourth most common cancer in terms of incidence and the fourth leading cause of cancer death in women. In 2020, an estimated 604,000 new cases and 342,000 deaths occurred worldwide (1). Early-stage CC (Ia1-Ib2, IIa1) has a favorable prognosis, with a 5-year survival rate of over 90% (2). However, the prognosis for locally advanced stages (IB3, IIa2, IIb-IVA) is not as promising, with a 5-year survival rate of only 50-70% (3). The prognosis is even worse for advanced stage CC with distant metastasis (IVb) or recurrence, with a 5-year survival rate of only 17% (4).

Genomic instability is one of the most common characteristics of human tumors. Cells in the human body experience tens of thousands of DNA damage incidents every day, and have evolved specialized DNA damage repair (DDR) mechanisms to maintain genomic stability (5). However, when DNA damage accumulates and leads to genomic instability, tumor development can occur, which is a hallmark event in cancer (6). Radiotherapy and chemotherapy are important treatments for CC, based on this tumor characteristic. In cancer treatment, radiotherapy mainly induces cancer cell death by ionizing radiation, causing DNA double-strand breaks (7). Platinum-based drugs primarily enter tumor cells and form Pt-DNA adducts to induce tumor cell death or apoptosis, producing anti-cancer effects (8, 9). However, the failure of radiotherapy and chemotherapy often occurs because tumor cells activate DDR responses through different signaling pathways, recognizing DNA damage induced by ionizing radiation or drugs, and conducting DNA repair, which leads to radiation resistance or chemotherapy resistance (10, 11).

Furthermore, DDR abnormalities not only lead to a high genomic mutation rate in tumor cells, but also cause changes in the tumor immune microenvironment, affecting the therapeutic efficacy of immune checkpoint inhibitors (ICIs) (12). Studies have shown that patients with DDR pathway mutations have a significantly higher average tumor mutation burden (TMB) than those without mutations, suggesting that DDR mutations may become a new biomarker for predicting the efficacy of ICIs (13). Among all DDR pathways, defects in the mismatch repair (MMR) pathway cause an increase in microsatellite instability, which has been shown to be related to the efficacy of ICIs, and has been approved by the US Food and Drug Administration as an indicator for predicting the efficacy of ICIs in cancer. The correlation between other DDR pathways and the efficacy of ICIs is also being explored (14). ICIs do not directly target DNA damage and repair, but increased DNA damage and abnormal repair can lead to enhanced genomic instability, changes in the tumor immune microenvironment, and affect the efficacy of ICIs (15). Therefore, exploring the characteristic genes that affect DDR, constructing a prognostic signature based on DDR-related characteristic genes, will help develop individualized monitoring and treatment plans for CC patients.

Compared to bulk RNA-seq, which detects RNA expression in all cells within a lesion, scRNA-seq detects RNA expression within individual cells. Therefore, the advantage of scRNA-seq is that it can reveal gene expression differences and higher resolution at the single-cell level, which provides a more favorable choice for exploring tumor cell heterogeneity. In this study, we integrated data obtained from second-generation sequencing technology and single-cell sequencing technology. We downloaded second-generation sequencing data from TCGA database of CC patients, and scRNA-seq GSE168652 dataset from GEO database, and selected GSE52903 chip dataset of CC patients as the validation set. We expect to complement the advantages of bulk RNA-sequence and scRNA-seq to explore genes that affect CC DDR heterogeneity more deeply and accurately, and then construct a signature to evaluate CC prognosis. In addition, we will explore the role of this signature in evaluating the immune microenvironment and tumor mutation burden, and evaluate patient drug sensitivity based on DDR, providing a reference for the clinical treatment of CC.

## 2 Materials and methods

### 2.1 Transcriptome data download and processing

The mRNA counts data obtained from bulk sequencing, and clinical data of patients with cervical squamous cell carcinoma and adenocarcinoma (CESC) were downloaded from the TCGA-CESC project and used as a training cohort for the study (https://portal.gdc.cancer.gov/). The FPKM values were transformed into transcripts per million (TPM) values. Based on the identification of the TCGA sample code 01, a total of 283 samples were retained from the original 309 samples for further bioinformatic analysis. Subsequently, the transcriptome and clinical data used as a validation cohort were obtained from GSE52903, which was downloaded from the Gene Expression Omnibus (GEO) database (https://www.ncbi.nlm.nih.gov/geo/). The GSE52903 dataset included RNA expression and clinical data from 56 CC patients. The clinical information of the patients in the training and validation groups has been organized and presented in tabular form in the supplementary materials. We transformed all data using log2 for subsequent analysis.

### 2.2 Single cell RNA sequencing data download and processing

The scRNA-seq dataset GSE168652 of CC was downloaded from the GEO database, containing scRNA-seq of CC tissue and normal adjacent tissue from a CC patient. Samples were integrated using the tSNE (t-Distributed Stochastic Neighbor Embedding) method in the R package “Seurat (version 4.3.0)”, and core cells were obtained by quality control filtering. Initial quality control filtering was defined using the criteria: ≤10% mitochondrial genes, ≤30% ribosomal genes, ≤5% hemoglobin genes, and 200~7000 genes/cell. We identified 3,000 highly variable genes and integrated samples using SCT correction. Then, by setting the “DIMS” parameter to 20, the tSNE method was used to reduce the dimension of the data, and the “KNN” method was used to set the resolution to 1.0 for cell clustering. The cells were annotated by cell surface markers through the R package “singleR (version 1.10.0)”. Afterward, the “PercentFeatureSet” function was applied to calculate the scores of DDR-related genes in the cell. The cells from GSE168652 were divided into high and low DDR groups based on the median of calculated DDR scores.

### 2.3 The acquisition of DDR related genes

Using “DNA damage repair” as a keyword, 10,526 genes related to DDR were retrieved from the Genecards database (http://www.genecards.org), of which 9,723 DDR-related genes with a correlation coefficient greater than 1.0 were selected.

### 2.4 Single sample gene set enrichment analysis

Performed single sample gene set enrichment analysis (ssGSEA) on samples in the TCGA-CESC cohort using the GSVA package (version 1.44.5) to obtain each sample’s DDR scores and the enrichment score of each immune cell. By defining an immune cell-associated gene set, the enrichment score of the gene set represents the density of tumor-infiltrating immune cells. Obtaining feature gene panels for every immune cell type was accomplished through previous publications (16). The results were plotted by ComplexHeatmap (version 2.12.1) package in R.

### 2.5 Weighted gene co-expression network analysis

The R package “WGCNA (version 1.71)” was used for weighted gene co-expression network analysis (WGCNA) in R. First, hierarchical clustering was performed on the TCGA-CESC cohort samples, outliers were detected and eliminated. Second, we used the function pickSoft Threshold to build a scale-free network. Thereafter, an adjacency matrix was built and transformed into a topological overlap matrix (TOM), and the gene dendrogram and module colors were built using the dissimilarity. Then correlations between modules and DDR phenotypes were then calculated using the WGCNA package. Modules with high correlation coefficients (*P*<0.05) were considered as candidate modules associated with DDR and were selected for subsequent analysis. In detail, the modules were constructed with the threshold value of the module dendrogram of 0.25, the outlier value of 170, and a minimum module size of 50 genes. Thus, we obtained the module genes that correlate highly with DDR. Correlation analysis was conducted using the Spearman correlation test.

### 2.6 Construction and validation of the prognostic signature associated with DDR

We performed univariate Cox analysis on the intersection genes of the gene sets related to DDR obtained from WGCNA analysis and the gene sets obtained from single-cell differential expression analysis. To avoid overfitting, the “glmnet (version 4.1-4)” package was used for Least Absolute Shrinkage and Selection Operator (LASSO) regression analysis to construct a prognostic DDR-related gene signature. According to the formula, a prognostic signature was constructed where the risk score was calculated as the sum of each gene’s expression value multiplied by its corresponding LASSO regression coefficient: risk score = exp-gene 1 × β1 + exp-gene 2 × β2 +… + exp-gene n × βn (where “exp-gene n” represents the expression value of gene n and “β” represents the corresponding coefficient). According to the calculation method of the obtained prognostic signature, the DDR scores of each patient enrolled in the study from the TCGA-CESC cohort was calculated and ranked. With the median DDR scores as the cutoff, all patients were allocated into low DDR group or high DDR group respectively. K-M analysis and ROC curves were used to evaluate the prognostic value of DDR-related signature. Patient survival curves were visualized by the R software “survminer (version 0.4.9)” package. The ROC curves were plotted using the “survivalROC (version 1.0.3)” package to evaluate the performance of the risk score in predicting 1, 2, 3, and 5-year overall survival (OS) in patients with CC. The DDR-related signature was validated using the external independent validation cohort GSE52903 to confirm its generalizability. Furthermore, a nomogram was developed that combined the DDR scores with clinical characteristics based on clinicopathological parameters. The nomogram model was plotted using the “cph” function in R to visualize the prediction model and predict the likely 1, 3, and 5-year patient mortality. The clinical utility of the nomogram was assessed using ROC curves and decision curve analysis (DCA).

### 2.7 Analysis of immune infiltration and mutation

Six kinds of immune-related algorithms (“CIBERSORT”, “EPIC”, “MCP_counter”, “xCell”, “TIMER”, “Quanti-seq”) were used to analyze the immune landscape between the high- and low-DDR groups. The association between the DDR scores and immune cells was assessed using Spearman’s correlation test. The Pearson correlation coefficient was used to examine the relationship between the DDR scores and the expression of the immune checkpoint genes. To further understand the differences in mutations between high- and low-DDR groups, the gene mutations in different groups was analyzed using the Maftools (version 2.12.0) package in R. cBioPortal (http://www.cbioportal.org/) is a database that integrates various genomic data types, from which we downloaded the mutation data of TCGA and performed mutation analysis on the high and low DDR groups. Next, we explored the characteristics of immune checkpoint-related genes in different groups with limma (version 3.52.4), ggplot (version 3.4.0), ggpubr (version 0.6.0), and ggExtra (version 0.1.0) packages. Top 20 genes with the highest mutation frequency were presented between different groups.

### 2.8 Gene set enrichment analysis

Gene set enrichment analysis (GSEA) was performed to identify enriched pathways associated with key genes to explore potential molecular mechanisms. We obtained GSEA software (version 3.0) from the GSEA website (http://software.broadinstitute.org/gsea/index.jsp). According to the expression level of genes in the signature, the cut-off value was the median value. The TCGA-CESC samples were divided into two groups: high and low expression group, all canonical pathways and the enriched gene sets in KEGG were selected for analysis. We grouped according to gene expression profiles and phenotypes with a minimum gene set of 5 and a maximum gene set of 5000. We performed one thousand replicate samples to assess relevant pathways and molecular mechanisms. Five significantly enriched terms or pathways in each group were selected and visualized.

### 2.9 Analysis of drug sensitivity

The pRRophetic (R package,version 0.5) was employed for drug sensitivity prediction, which utilized ridge regression to estimate the half-maximal inhibitory concentration (IC50) for each patient from TCGA-CESC. The prediction accuracy was assessed using 10-fold cross-validation with the Genomics of Drug Sensitivity in Cancer (GDSC, https://www.cancerrxgene.org/) training set. The correlation between DDR-related signatures and the sensitivity of some antitumor drugs was displayed by ggplot2 (17). Pearson correlation analysis was conducted to explore the correlations between different DDR groups and drug sensitivity.

### 2.10 Cell culture and transfection

HeLa cells derived from human cervical adenocarcinoma were obtained from the cell bank of Hunan Normal University, Changsha, China. ECT1 cells derived from human normal cervix were purchased from the Qingqi (Shanghai, China) Biotechnology Development Co. HeLa and ECT1 were cultured in DMEM medium (Procell, Wuhan, China) supplemented with 1% penicillin-streptomycin (BI, Israel) and 10% fetal bovine serum (Procell, Wuhan, China). CaSki cells derived from human cervical squamous carcinoma were purchased from the Fenghui (Changsha, China) Biotechnology Development Co., and were cultured in RPMI-1640 medium under the same conditions at 37°C and 5% CO_2_. Collect cells in logarithmic growth phase for subsequent experiments. Cells were transfected with the previously synthesized sh-*ITGB1* (Genechem Inc, Shanghai, China) using the Lipo8000™ Transfection Reagent (Beyotime Biotechnology, Shanghai, MA, China) according to the manufacturer’s protocol. Subsequent experiments were performed 48 hours after transfection of the cells. The shRNA sequences for *ITGB1* are provided in **Supplementary Table S1**.

### 2.11 Quantitative real-time polymerase chain reaction

We used quantitative real-time polymerase chain reaction (qRT-PCR) to detect the expression levels of *ITGB1*, *ZC3H13* and *TOMM20* in ECT1, CaSki and HeLa cells, as well as to assess the effectiveness of the synthesized sh-*ITGB1* in inducing knockdown. According to the manufacturer’s instructions, total RNA was isolated using Trizol reagent (Vazyme, RNA isolater Total RNA Extraction Reagent, Nanjing, China). Reverse transcription and qRT-PCR were performed using PerfectStart Uni RT&qPCR Kit (TransGen Biotech, Beijing, China). The 2^−ΔΔCt^ method was employed to calculate the relative expression of genes, with normalization to glyceraldehyde-3-phosphate dehydrogenase (GAPDH), respectively. Primers were synthesized by Sangon Biotect Inc (Shanghai, China), and their sequences were listed in **Supplementary Table S2**. All data are presented as the mean ± SD of three independent experiments.

### 2.12 Scratch wound healing assay

Scratch wound healing assays were performed in CaSki and HeLa cells transfected with sh-*ITGB1* or transfected control group. After CaSki and HeLa reached 90-100% in 6-well culture plates, a line within CaSki and HeLa was scraped using a sterile plastic pipette tip in each culture well. After washing with PBS two times, cells were further cultured in DMEM/1640 medium supplemented with 10% FBS at 37 °C. The scraped wounds were photographed under a microscope at 0 and 24 hours. The pictures were then analyzed using Image J software.

### 2.13 Migration and invasion assays

For migration assay, CaSki and HeLa were diluted to 1× 10^5^/mL with serum‐free medium, 200μL cell suspension was added to the upper transwell chamber, and 800μL medium containing 10% fetal bovine serum was added to the lower chamber, respectively. The upper chamber was carefully immersed in the lower chamber liquid with sterile forceps. The 24‐well plate with transwell chamber was incubated at 37°C for 24 hours. The liquid was removed from the upper chamber after 24 hours and washed three times with PBS. After crystal violet staining, the upper chamber was observed under the electron microscope and photographed. For invasion assay, Matrigel (BD Biosciences, Franklin Lakes, NJ, USA) was coated into transwell chamber and placed in the oven at 37℃ for 2 hours. Then following steps were the same as the migration experiment. Each experiment was repeated three times. All data were presented as the means ± SD of three independent experiments.

### 2.14 Comet assay

After incubation, the cells were collected and resuspended using ice-cold PBS. 1 × 10^5^/ml cells were mixed with 0.7% low-melt agarose at 37°C at a ratio of 1:7.5 (v/v) and immediately pipetted onto frosted glass slides. To perform neutral comet assay, the glass slides were placed in the neutral lysis buffer (add 1ml of DMSO every 9ml before use) at 4°C for 2 h, washed three times in PBS, and placed in freshly prepared alkaline electrophoresis solution (1mmol/L EDTA, 300mmol/L NaOH) for 30 min at 37°C. The slides were treated with 25 V (1 V/cm) electrophoresis and 300 mA for 25 min. The slides were then neutralized to pH 7.5 in Tris-HCl buffer and stained for 20 min with DAPI (2.5 µg/ml) in the dark. The images were viewed under a fluorescence microscope and further analyzed by OpenComet. According to the methods described previously (18), we included 100 cells in each group for statistical analysis.

### 2.15 Immunohistochemistry

The protein abundance of ITGB1 in CC tissues and normal cervical tissues was detected using IHC. Primary antibody against ITGB1 were purchased from ImmunoWay Biotechnology (SuZhou, China). Tissue samples were fixed with 4% paraformaldehyde and processed using standard procedures of dehydration, fixation, embedding, and slicing. The slides were then treated with primary antibodies at 4°C overnight. Prior to incubating with the secondary antibody, the slides were washed three times with PBS. The PV-9000 Kit (Zsbio, Beijing, China) was used as the secondary antibody, and the slides were incubated for 1 hour at room temperature, protected from light. The substrate diaminobenzidine (DAB, Beyotime) was used for antibody detection, and the slides were counterstained with hematoxylin (Beyotime). The cell nucleus was stained blue by hematoxylin. The IHC images were obtained using a Zeiss light microscope and analyzed for mean optical density using Image J software.

### 2.16 Statistical analyses

Statistical analysis was calculated using the Student’s t-test when comparing two groups in cytology experiments. The correlation analysis between variables was conducted using both Spearman or Pearson methods. Kaplan-Meier analysis was applied to assess the difference of overall survival between the high and low DDR groups. The statistical analysis is carried out using GraphPad Prism (version 8.0) and R software (version 4.2.0). The P-values <0.05 are considered statistically significant.

## 3 Results

### 3.1 Schematic diagram of the study design

**Figure 1:**
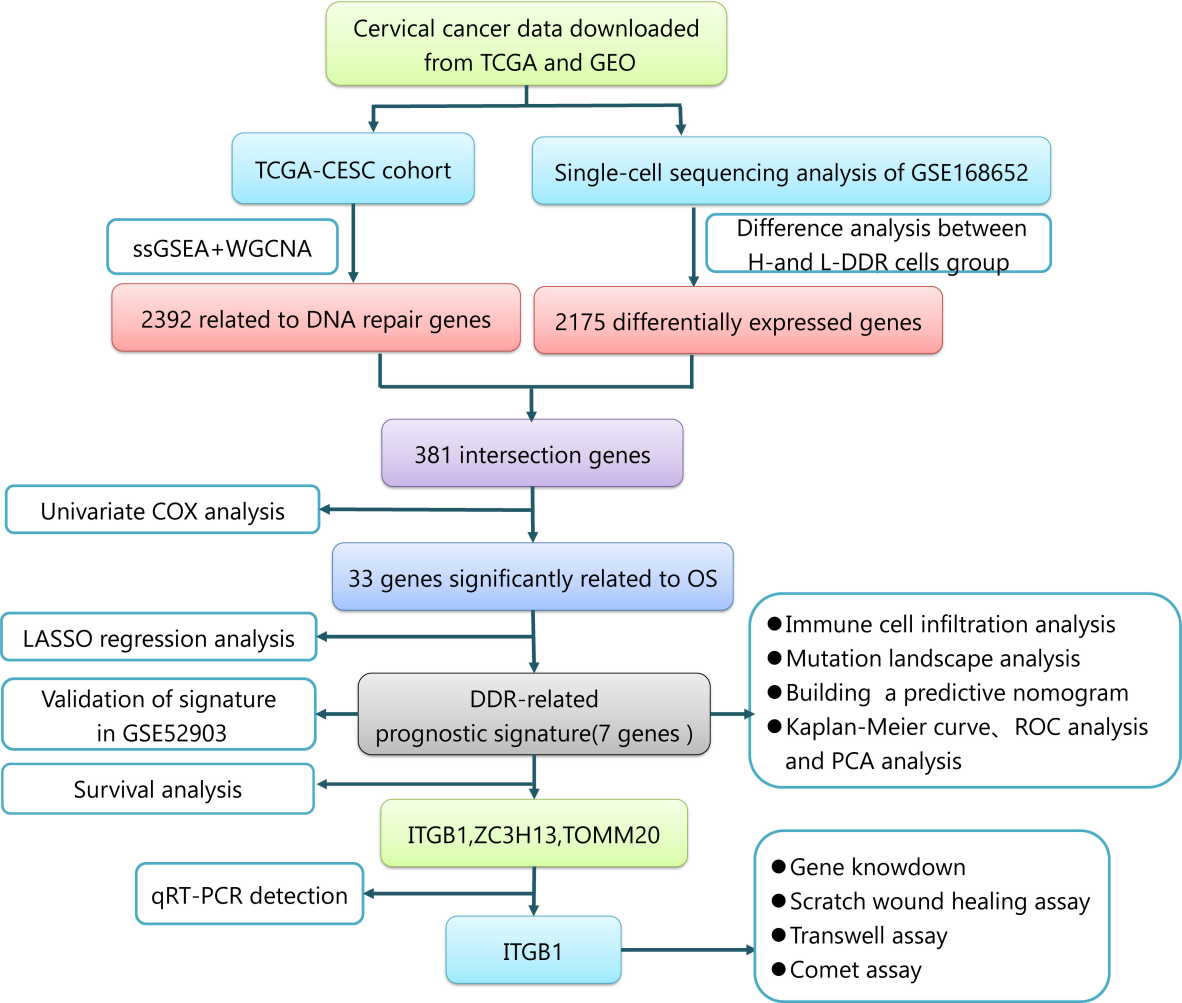
The entire work flowchart of this research.

**Figure 1** displays the flowchart of the entire work.

### 3.2 Single cell RNA sequencing data analysis

**Figure 2:**
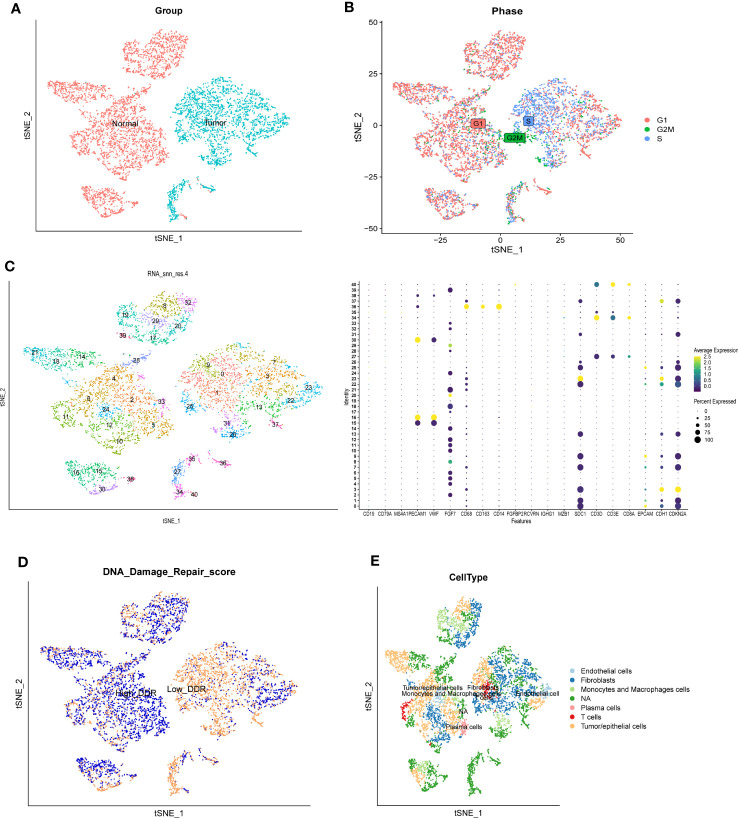
Single-cell RNA sequencing analysis of GSE168652. **(A)** The two samples were integrated, and the results showed that the two samples had no obvious batch effect. **(B)** Cell cycle genes could be effectively clustered together, exhibiting no apparent distinction and facilitating subsequent analysis. **(C)** Dimensionality reduction and cluster analysis. All cells in 2 samples were clustered into 41 clusters. **(D)** The percentage of DDR genes in each cell. The cells were divided into high- and low-DDR cells. **(E)** According to the surface marker genes of different cell types, the cells are annotated as endothelial cells, fibroblasts, plasma cells, T cells, monocytes and macrophages cells, and tumor/epithelial cells, respectively.

We first analyzed the single-cell sequencing dataset of CC to integrate different samples, and tSNE analysis showed that there was no obvious batch effect of the two samples, as shown in **Figure 2A**. Cell cycle genes could be effectively clustered together, exhibiting no apparent distinction and facilitating subsequent analysis (**Figure 2B**). Then, we clustered all cells into 41 clusters using the k-nearest neighbor (KNN) clustering algorithm (**Figure 2C**). Next, we input 9723 DDR-associated genes using the “PercentFeatureSet” function, and obtained the scores of DDR-associated genes for each cell. The cells were classified into two groups, low DDR cells and high DDR cells, according to the median DDR scores and presented in a tSNE plot (**Figure 2D**). Based on the surface marker genes of different cell types (**Supplementary Table S3**), we observed their expression in different clusters (**Figure 2C**), and identified six cell types, including endothelial cells, fibroblasts, plasma cells, T cells, macrophages and tumor/epithelial cells (**Figure 2E**). Finally, we identified 2175 genes by analyzing the differentially expressed genes between high and low DDR group of cells.

### 3.3 ssGSEA and WGCNA

**Figure 3:**
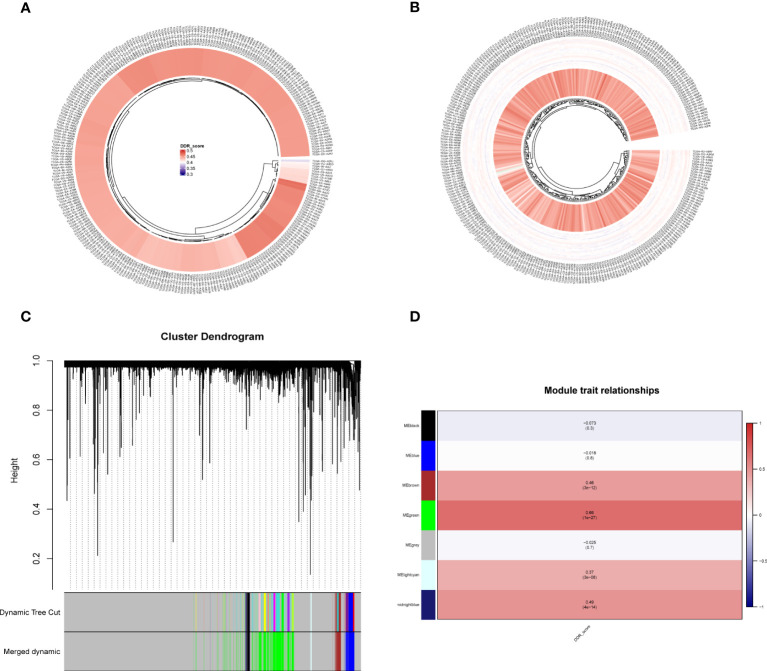
ssGSEA analysis and DDR-related genes were screened by WGCNA. **(A)** ssGSEA calculates DDR scores in TCGA-CESC cohort. **(B)** The immune infiltration levels were quantified using ssGSEA in the TCGA-CESC cohort. **(C, D)** WGCNA found that MEgreen, MEbrown, MElightcyan, MEmidnightblue, MEblack, and MEblue modules were closely related to the scores of DDR.

DDR scores were calculated for each sample in the TCGA cohort using ssGSEA (**Figure 3A**). Then, the ssGSEA method was used to quantify the TCGA-CESC samples to assess the infiltration level of immune cells (**Figure 3B**). In the TCGA cohort, gene modules associated with DDR phenotype were obtained by WGCNA of 283 samples. A total of 6 non-gray modules were obtained by setting the module dendrogram of 0.25, the outlier value of 170, and a minimum module size of 50 genes (**Figure 3C**). We found that MEgreen, MEbrown, MElightcyan, MEmidnightblue, MEblack, and MEblue were closely related to the DDR scores in the non-gray modules (**Figure 3D**). The grey module contains all genes that were not included in the clustering and is therefore an invalid module that should not be used in subsequent analyses. The MEbrown, MEgreen, MElightcyan, and MEmidnightblue modules had the high correlation (*P*<0.05) with the DDR. Therefore, we chose them for the follow up analysis.

### 3.4 Construction and validation of DDR-related prognostic signature

**Figure 4:**
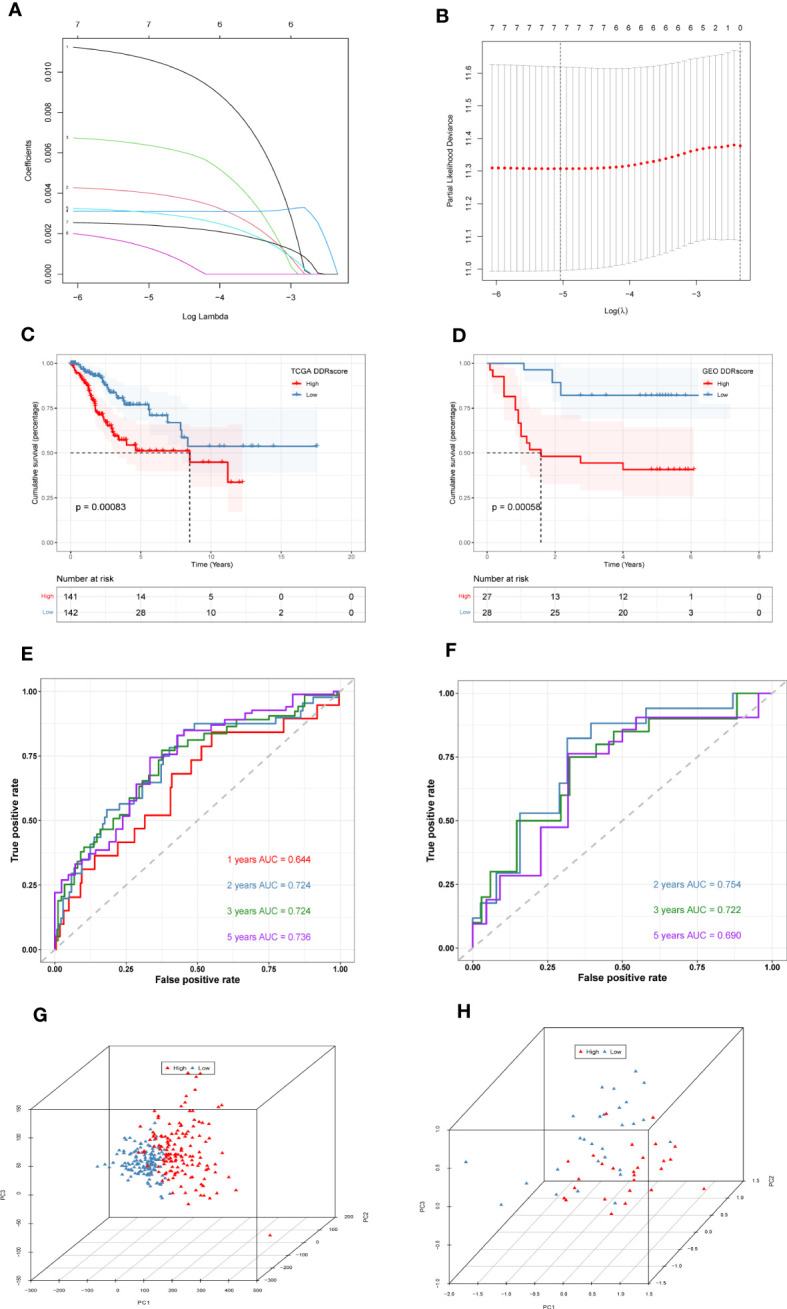
Construction and validation of DDR-related prognostic signature. **(A)**LASSO coefficient profiles of the seven genes *EFEMP2*, *TPM3*, *ZC3H13*, *ITGB1*, *TOMM20*, *ROCK2*, and *TCP1* in the TCGA cohort. **(B)** A coefficient profile plot was generated against the log (lambda) sequence. Selection of the optimal parameter (lambda) in the LASSO model. **(C)** Survival analysis of TCGA cohort. The prognosis was significantly worse in the high-DDR group (*P*<0.001). **(D)** Survival analysis of GSE52903 Cohort. The prognosis was significantly worse in the high-DDR group (*P*<0.001). **(E)** ROC curve of TCGA cohort. The AUC values of the signature in 1, 2, 3, and 5 years were 0.644, 0.724, 0.724, and 0.736, respectively. **(F)** ROC curve of GSE52903 cohort. The AUC values of the signature in 2, 3, and 5 years were 0.745, 0.722, and 0.690, respectively. **(G, H)** PCA analysis in TCGA cohort and GSE52903 cohort. It was found that the signature could group CC patients well in both the training and validation cohorts.

First, we collected 381 genes (**Supplementary Table S4**) from the intersection of differentially expressed genes obtained from single-cell sequencing data analysis and DDR-associated genes obtained from WGCNA for subsequent analysis. In the TCGA cohort, 33 genes (**Supplementary Table S5**) associated with patient prognosis were initially obtained by univariate COX analysis at *P* < 0.05. Afterward, lasso regression was performed, and the results showed that gene contraction stabilized when the number of included genes was 7 (**Figures 4A, B****)**. These 7 genes were *EFEMP2*, *TPM3*, *ZC3H13*, *ITGB1*, *TOMM20*, *ROCK2*, and *TCP1* (**Table 1**). The prognostic signature constructed by these 7 genes was calculated as follows:

**Table 1:** Seven genes were identified by Lasso regression to construct a prognostic signature.

Gene	HR	P-value
EFEMP2	1.017	<0.05
TPM3	1.010	<0.05
ZC3H13	1.031	<0.05
ITGBI	1.008	<0.05
TOMM20	1.005	<0.05
ROCK2	1.029	<0.05
TCP1	1.007	<0.05

DDR=EFEMP2*0.01065267683731472+TPM3*0.004023509277444922+ZC3H13*0.006397321057257991+ITGB1*0.0031003552748523713+TOMM20*0.0030558758870910664+ROCK2*0.0013326606915910497+TCP1*0.0024293569783406395. The median signature score was used to classify patients into high- or low-DDR groups. Kaplan–Meier analysis revealed that patients with high DDR scores suffered worse outcomes in the TCGA training cohort (*P* < 0.05, **Figure 4C**). Similarly, in the GSE52903 validation cohort, we also observed that the prognosis of high DDR patients was worse than that of low DDR patients (*P* < 0.05, **Figure 4D**). To further explore the accuracy of DDR-related signature in the prognostic assessment of CC patients, we performed ROC curve analysis in both the training cohort and the validation cohort. As shown in **Figure 4E**, in the TCGA cohort, the areas under the curve (AUC) values were 0.644, 0.724, 0.724, and 0.736 for 1, 2, 3, and 5 years, respectively. In the validation cohort, we found that the regions under the curve at 2, 3, and 5 years were 0.754, 0.722, and 0.690, respectively (**Figure 4F**), indicating that DDR-related prognostic signature has high accuracy in predicting the prognosis of patients in both cohorts. Finally, PCA analysis was performed in the training and validation set signatures, respectively, it was found that the signature could group CC patients well in both the training cohort and the validation cohort (**Figures 4G, H****)**.

### 3.5 Immune infiltration analysis and mutation landscape

**Figure 5:**
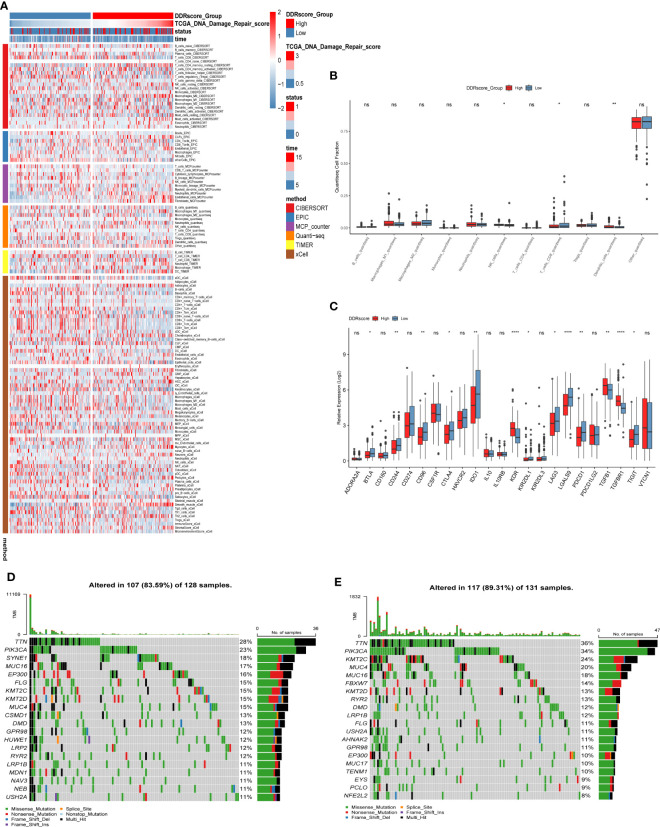
Immune infiltration analysis and mutation landscape in high and low DDR groups. **(A)** Heat map of immune cell infiltration in high and low DDR groups. **(B)** Expression of immune cells in the high and low DDR groups. **(C)** Expression of immune checkpoint-related genes in high and low DDR groups. **(D)** Mutation in the high DDR group. **(E)** Mutations in the low DDR group. *P < 0.05, **P < 0.01, ****P < 0.0001; ns, not significant.

To understand the immune microenvironment in different DDR status, we explored the immune infiltration levels between the high- and low-DDR patient groups using six methods. The results showed that immune cell infiltration was higher in the low DDR group, including macrophage M1, plasma, T and B cells, as shown in **Figure 5A**. Then, we investigated the expression of different immune cells in the high or low DDR groups, and we found that CD8+ T cells were highly expressed in the low DDR group, while NK cells and dendritic cells were highly expressed in the high DDR group (**Figure 5B**). After that, the expression of genes related to immune checkpoints were investigated, as shown in **Figure 5C**, and most of the immune checkpoint-related genes, such as *IDO1*, *CD244*, and *LAGLS9*, were found higher expression levels in the low DDR group compared to the high DDR group. Subsequently, we analyzed the mutations of the top 20 mutated genes in the high and low DDR groups, respectively. The results showed that the mutation incidence of the top 20 mutated genes in the high or low DDR groups was 83.59% and 89.31%, respectively (**Figures 5D, E****)**. The differentially mutated genes in the two groups were compared shown in **Supplementary Figure S1**. Finally, we analyzed the mutations in 7 genes in the signature (**Supplementary Figure S2**).

### 3.6 Cell localization of 7 modeling genes

**Figure 6:**
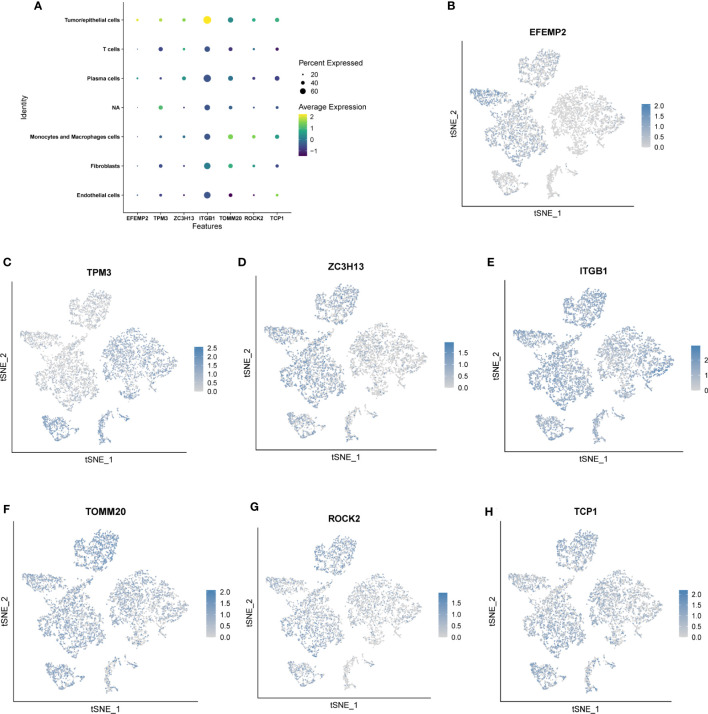
Location and expression of 7 genes in prognostic signature at the single-cell level using tSNE plots. **(A-H)** *EFEMP2* was mainly expressed in plasma cells as well as in tumor/epithelial cells, *TPM3* was mainly expressed in T cells, *ZC3H13* was mainly expressed in plasma cells, *ITGB1* was mainly expressed in tumor/epithelial cells and plasma cells, *TOMM20* was mainly expressed in macrophages and tumor/epithelial cells, *ROCK2* was mainly expressed in tumor/epithelial cells, and *TCP1* was expressed in plasma cells.

We used single-cell sequencing data to investigate the expression of modeling genes in different cell types. As shown in **Figures 6A–H**, *EFEMP2* was mainly expressed in plasma cells as well as in tumor/epithelial cells, *TPM3* was mainly expressed in T cells, *ZC3H13* was mainly expressed in plasma cells, *ITGB1* was mainly expressed in tumor/epithelial cells and plasma cells, *TOMM20* was mainly expressed in macrophages and tumor/epithelial cells, *ROCK2* was mainly expressed in tumor/epithelial cells, and *TCP1* was expressed in plasma cells.

### 3.7 Nomogram construction and survival analysis

**Figure 7:**
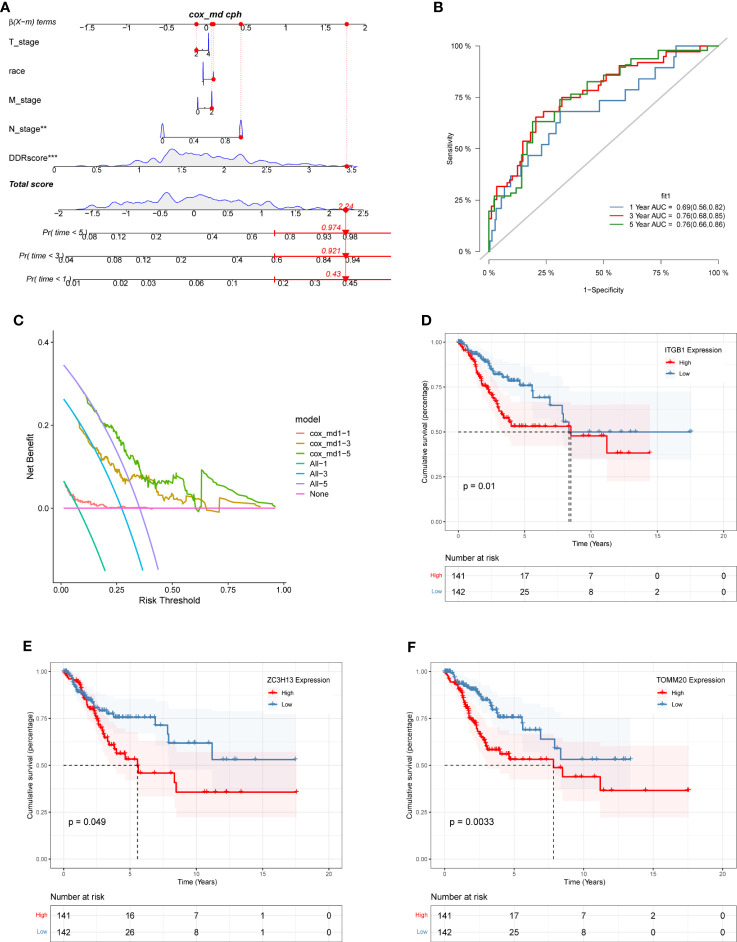
The construction of a nomogram and survival analysis. **(A)** Constructed nomogram based on the race, T stage, N stage, M stage, and DDR scores of patients in TCGA. The estimated mortality rates for patients at 1, 3, and 5 years were 0.43, 0.921, and 0.974. **(B)** ROC curve of the nomogram. The area under the curve (AUC) at 1, 3, and 5 years were 0.69, 0.76, and 0.76, respectively. **(C)** Decision curve analysis. The effect of this nomogram was superior to other clinical indicators. **(D-F)** The survival analysis results demonstrated: *ITGB1 ***(D)**, *ZC3H13 ***(E)**, and *TOMM20 ***(F)** were identified as independent risk factors influencing survival outcomes among the prognostic gene signature.

A nomogram combining clinical data and DDR scores was constructed to confirm further whether the DDR-related gene signature could serve as an independent prognostic factor for CC. The nomogram was created based on the race, T stage, N stage, M stage, and DDR scores of patients in the TCGA database, and the mortality rates of patients at 1, 3, and 5 years were estimated to be 0.43, 0.921 and 0.974 (**Figure 7A**). To assess the accuracy of this nomogram further, a ROC prognostic analysis was performed. The results showed that the area under the curve (AUC) was 0.69, 0.76, and 0.76 at 1, 3, and 5 years, respectively (**Figure 7B**). We also performed a decision curve analysis to assess the clinical decision values by calculating the area of each clinical feature and the horizontal axis of none. The findings of the decision curve analysis indicated that our nomogram might perform better than other clinical indicators in predicting the survival of CC patients (**Figure 7C**). After COX regression and lasso regression analysis, we screened seven genes and performed survival analysis for each of these seven genes (**Supplementary Figure S3**). The results showed that the prognosis of patients with high expression of *ITGB1*, *ZC3H13* and *TOMM20* was significantly worse than those with low expression (*P*<0.05, **Figures 7D–F**).

### 3.8 HPA and GSEA analysis

**Figure 8:**
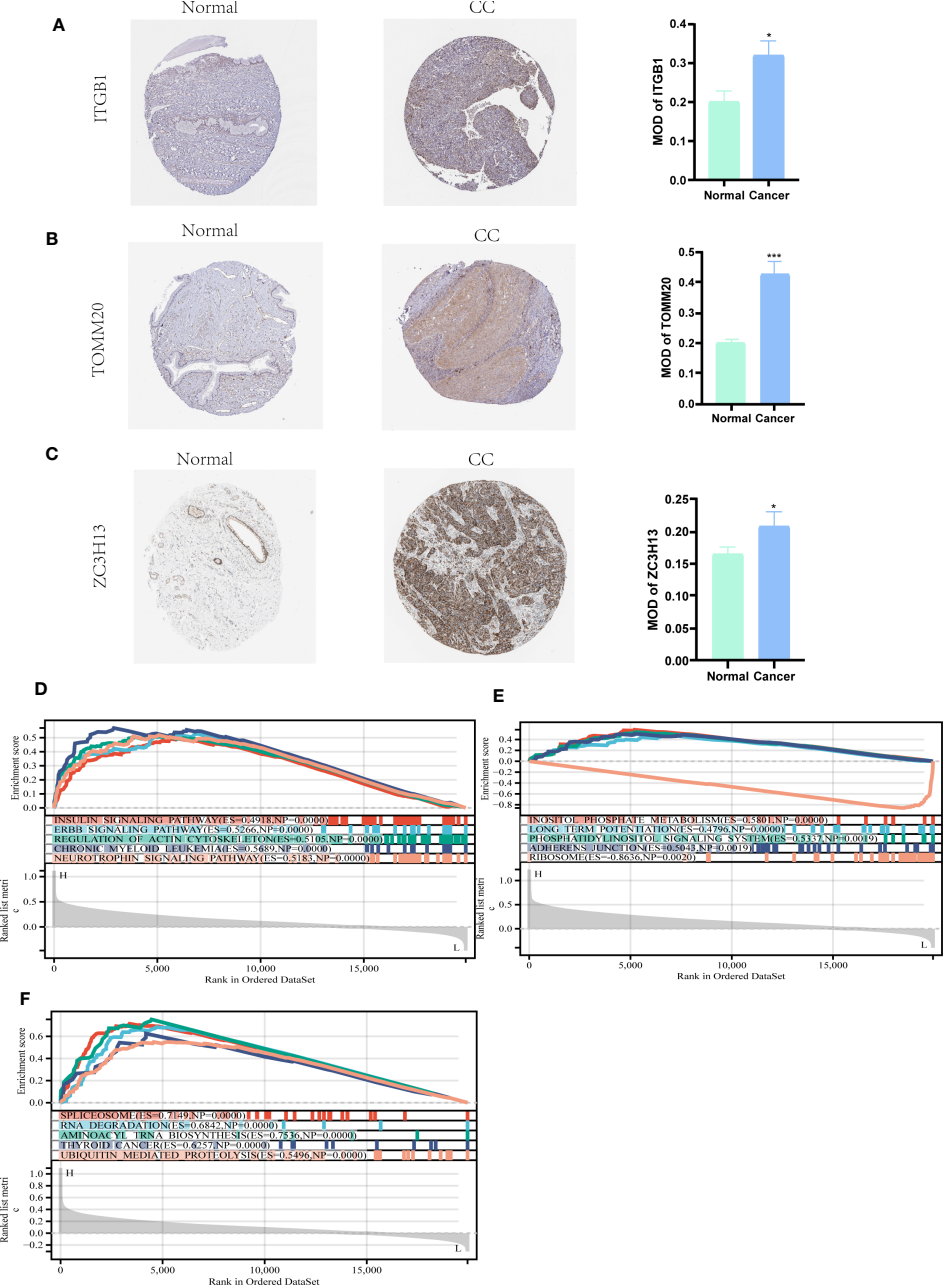
HPA and GSEA analysis. **(A-C)** Compared with normal cervical tissues, ITGB1, ZC3H13, and TOMM20 were more highly expressed in CC tissues (*P*<0.05). **(D)** The top five most activated KEGG terms involving *ITGB1* were insulin signaling pathway, ERBB signaling pathway, regulation of actin cytoskeleton, chronic myeloid leukemia, and neurotrophin signaling pathway. **(E)** The top five most activated KEGG terms involving *ZC3H13* were inositol phosphate metabolism, long term potentiation, phosphatidylinositol signaling system, adherens junction, and ribosome. **(F)** The top five most activated KEGG terms involving *TOMM20* were spliceosome, RNA degradation, aminoacyl trna biosynthesis, thyroid cancer, and ubiquitin mediated proteolysis. *P < 0.05, ***P < 0.001.

We further used the Human Protein Atlas Database (www.proteinatlas.org) to visually assess the expression of ITGB1, ZC3H13, and TOMM20 in CC tissues and normal cervical tissues. The results showed that ITGB1, ZC3H13, and TOMM20 were expressed at higher levels in CC tissues than in normal cervical tissues (**Figures 8A–C**). These three hub genes were subjected to GESA analysis separately, and the results are shown in the figure. The top five most activated Kyoto Encyclopedia of Genes and Genomes (KEGG) terms involving *ITGB1* were insulin signaling pathway, ERBB signaling pathway, regulation of actin cytoskeleton, chronic myeloid leukemia, and neurotrophin signaling pathway (**Figure 8D**). The top five most activated KEGG terms involving *ZC3H13* were inositol phosphate metabolism, long term potentiation, phosphatidylinositol signaling system, adherens junction, and ribosome (**Figure 8E**). The top five most activated KEGG terms involving *TOMM20* were spliceosome, RNA degradation, aminoacyl trna biosynthesis, thyroid cancer, and ubiquitin mediated proteolysis (**Figure 8F**).

### 3.9 Drug sensitivity analysis

**Figure 9:**
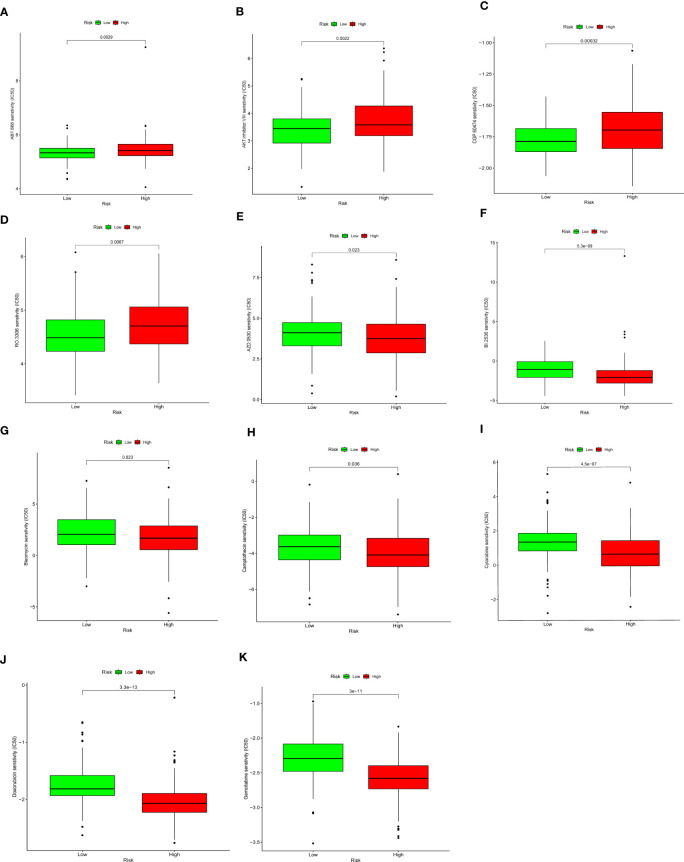
Drug sensitivity analysis. Low DDR group presented higher sensitivity to Veliparib (ABT.888) **(A)**, AKT inhibitor VIII **(B)**, CGP.60474 **(C)**, and RO.3306 **(D)**, and lower sensitivity to Saracatinib(AZD.0530) **(E)**, BI.2536 **(F)**, Bleomycin **(G)**, Camptothecin **(H)**, Cytarabine **(I)**, Doxorubicin **(J)**and Gemcitabine **(K)**.

The “pRRophetic” algorithm was applied to evaluate the sensitivity to antineoplastic drugs in CC patients with different DDR status (**Figure 9**). The analysis showed that the IC50 values of DDR-related drugs such as Veliparib (ABT.888), AKT inhibitor VIII, CGP.60474, and RO.3306 were higher in the high DDR group than in the low DDR group (**Figures 9A–D**). The IC50 values of Saracatinib(AZD.0530), BI.2536, Bleomycin, Camptothecin, Cytarabine, Doxorubicin and Gemcitabine were higher in the low DDR group than in the high DDR group (**Figures 9E–K**).

### 3.10 Differential expression of three hub genes in HeLa, CaSki and ECT1 cells

**Figure 10:**
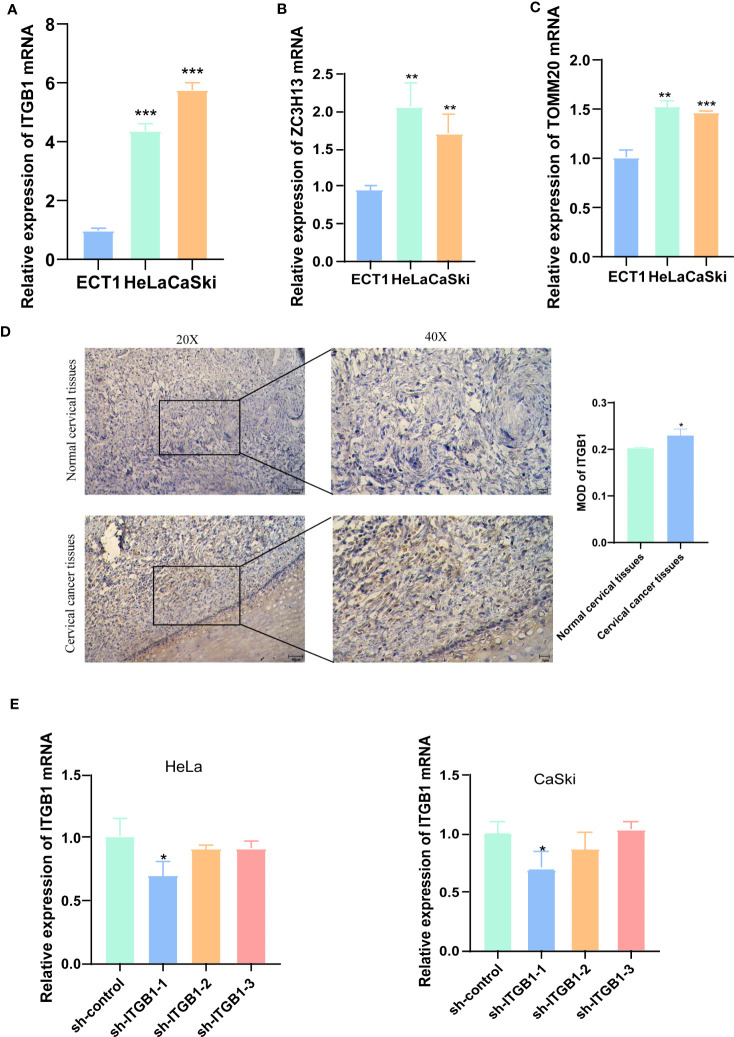
IHC and qRT-PCR results. **(A-C)** qRT-PCR detected the mRNA levels of *ITGB1*, *ZC3H13*, and *TOMM20* in HeLa, CaSki and ECT1 cells. **(D)** IHC results showed that ITGB1 was highly expressed in CC tissues compared with normal cervical tissues. **(E)** qRT-PCR evaluated the level of *ITGB1* mRNA 48h after transfection. And sh-*ITGB1*-1 had a better knockdown potency, which was used in further *in vitro* experiments. *P < 0.05, **P < 0.01, ***P < 0.001.

We used qRT-PCR to detect the mRNA levels of *ITGB1*, *ZC3H13*, and *TOMM20* in HeLa, CaSki and ECT1 cells (**Figures 10A–C**). We found that all three genes were highly expressed in HeLa and CaSki cells compared to ECT1 cells. *ITGB1* has the most obviously ratio of expression in CaSki and HeLa to ECT1 among three genes, so we selected *ITGB1* for subsequent analysis. Subsequently, the results of IHC sections also showed higher protein level of *ITGB1* in CC tissues (**Figure 10D**).

### 3.11 *ITGB1* knockdown reduces HeLa and CaSki cell migration and invasion *in vitro*

**Figure 11:**
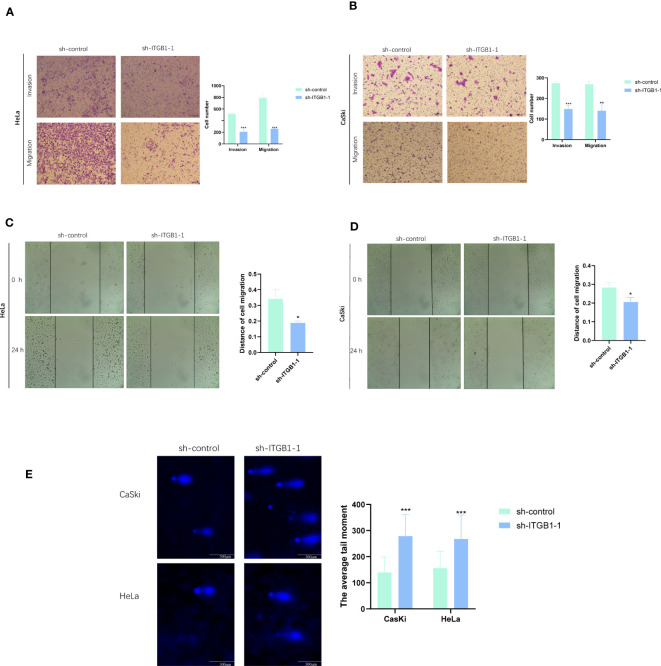
Functional exploration and expression validation of *ITGB1*. **(A, B)** Transwell assay. The migration and invasion capacity of HeLa and CaSki cells decreased significantly after the *ITGB1* knockdown. **(C, D)** Scratch wound healing assay. A significantly slower wound healing rate was observed in cells with a decreased expression of the *ITGB1* gene. **(E)** Comet assay. The DDR capacity of HeLa and CaSki cells decreased significantly after the *ITGB1* knockdown. All data were presented as the means ± SD of three independent experiments. *P < 0.05, **P < 0.01, ***P < 0.001.

To assess the ability of shRNA knockdown of gene *ITGB1* in HeLa and CaSki cell lines, we assessed *ITGB1* mRNA levels after transfected for 48 hours using a qRT-PCR method (**Figure 10E**). We found that sh-*ITGB1*-1 could reduce *ITGB1* mRNA expression levels (*P*<0.05) and could be used for further *in vitro* experiments. The migration and invasion ability of HeLa and CaSki cells were significantly decreased after *ITGB1* knockdown. We found that the percentage of cells migrating through the transwell plate significantly decreased after shRNA knockdown (**Figures 11A, B**). Also, scratch assay results showed that migration ability of HeLa and CaSki cells was drastically reduced when *ITGB1* was knockdown (**Figures 11C, D**).

### 3.12 *ITGB1* knockdown reduces the DDR ability of HeLa and CaSki cells *in vitro*

In comet assay, the lengthening of cells trails means the weaker DDR ability. The results showed that after knockdown of *ITGB1*, the tailing of HeLa and CaSki cells was prolonged, suggesting that *ITGB1* can weaken the DDR of HeLa and CaSki cells (**Figure 11E**).

## 4 Discussion

In this study, we constructed a DDR-related signature composed of 7 genes, including *EFEMP2*, *TPM3*, *ZC3H13*, *ITGB1*, *TOMM20*, *ROCK2*, and *TCP1*. We explored the differences in prognosis, immune microenvironment, mutation status, and drug sensitivity between high and low DDR groups based on this signature. We found three key prognostic genes in the signature, *ITGB1*, *ZC3H13*, and *TOMM20*, and selected the key gene *ITGB1* to verify its impact on the proliferation, migration, invasion, and DDR capacity of CaSki and HeLa cells.

Due to the tremendous progress in microarray technology and second-generation sequencing technology, many prognostic models have been developed to predict the prognosis of cancer patients. Unfortunately, there is currently a lack of biomarkers or models that can accurately predict the prognosis of CC (19, 20). We used single-cell clustering analysis and second-generation sequencing technology to obtain a DDR-related prognostic signature through comprehensive analysis. DNA damage response refers to a series of reactive events to genomic DNA damage, including DNA damage detection, DDR pathway, and cell fate determination (21, 22). DDR is part of the DNA damage response, and based on different types of DNA damage, DDR initiates the repair process through different pathways (23). According to our DDR scores signature, patients with low DDR have a higher survival rate than those with high DDR, providing a basis for further accurate prognostic judgments. The AUC values for 1 year, 2 years, 3 years, and 5 years in the TCGA cohort were 0.644, 0.724, 0.724, and 0.736, respectively. The ROC curve shows that our prognostic signature has good accuracy. At the same time, the robustness of this signature was validated in GEO datasets. The results showed that in the GEO external validation set, the OS of the high DDR group was significantly lower than that of the low DDR group, and the AUC values for 2 years, 3 years, and 5 years were 0.754, 0.722, and 0.690, respectively. In addition, by combining T staging, N staging, M staging, race, and DDR scores, a line chart survival signature was established to predict 1/3/5-year survival rates. The results showed that DDR scores can be used as an indicator for predicting patient survival rate and is superior to TNM staging, which has important guiding significance for clinical practice.

The 7 genes included in our signature are *ITGB1*, *ZC3H13*, *TOMM20*, *EFEMP2*, *TPM3*, *ROCK2*, and T*CP1*. Integrin-β (ITGB) is a member of the integrin superfamily and plays a crucial role in cell adhesion, proliferation, and differentiation (24). Previous studies have found that *ITGB1* inhibits radiosensitivity and enhances DDR in head and neck squamous cell carcinoma, pancreatic cancer and lung cancer (25–27). However, no association between *ITGB1* and DDR has been found in CC. *ZC3H13* is located on human chromosome 13q14.13 (28) and mainly promotes CC stemness and chemoresistance by affecting N6-methyladenosine and mRNA methylation (29). *TOMM20* is a receptor and a critical subunit of the outer mitochondrial membrane translocase complex (TOM complex). Studies have shown that *TOMM20* expression directly affects mitochondrial function, including ATP production, membrane potential maintenance, and regulation of tumor cell activity such as S phase cell cycle and apoptosis (30). *EFEMP2*, also known as fibulin-4, is a member of the fibulin family (31). High expression of *EFEMP2* in CC is associated with lymph node metastasis and poor prognosis, and may promote angiogenesis (32). TPM is a filamentous actin-binding protein that can bind to actin. *TPM3* is an important member of the TPM family and stabilizes the cell skeleton microfilaments (33). Studies on glioma, colon cancer, and liver cancer have shown that *TPM3* affects tumor occurrence and development through gene fusion and epithelial-mesenchymal transition (EMT), and has high expression levels (34). *ROCK2* is a key signaling molecule in the Rho/ROCK signaling pathway and plays an essential role in regulating gene expression by regulating the activity or phosphorylation of target proteins (35). Studies have shown that *ROCK2* is critical for cancer cell migration and invasion (36). *TCP1* is one of the subunits of the chaperonin-containing TCP-1 (CCT) complex and participates in protein folding, cell proliferation, apoptosis, cell cycle regulation, and drug resistance (37). *TCP1* is a factor that leads to breast and ovarian cancer resistance, and upregulation of *TCP1* can promote CC progression (38–40).

Three genes, *ITGB1*, *ZC3H13*, and *TOMM20*, included in the signature can independently predict prognosis. qRT-PCR results showed that all three genes were expressed at intermediate to high levels in CaSki and HeLa cells compared to immortalized cervical squamous cells ECT1. Additionally, analysis of the HPA database showed that the protein levels of *ITGB1*, *ZC3H13*, and *TOMM20* were high in CC tissues. *ITGB1* has the most obviously ratio of expression in CaSki andHeLa to ECT1 among three genes, so we selected *ITGB1* for subsequent analysis. The results of IHC also proved that *ITGB1* was highly expressed in CC tissue, compared with non-cancer tissues. Cell experiments verified that knockdown of *ITGB1* in CaSki and HeLa cells could significantly inhibit cancer cell proliferation, migration, and invasion abilities. Furthermore, comet assay results showed that knockdown of *ITGB1* could inhibit DDR ability in CaSki and HeLa cells. This provides a potential therapeutic target for CC.

As our understanding of cancer treatment mechanisms increases, more and more researches indicate the involvement of DDR in anti-tumor immune responses (15, 41, 42). Our study found that compared with the high DDR group, the low DDR group had stronger immune cell infiltration, with immunosuppressive cells such as NK cells and M2 macrophages enriched in the high DDR group, leading to poorer prognosis, while the activated T cells and B cells were more abundant in the low DDR group, resulting in a better prognosis. Moreover, in gastric cancer and melanoma, it has also been found that patients with low DDR scores have significantly increased immune infiltration, consistent with our results (43, 44). Studies have shown that changes in tumor DDR pathways are significantly correlated with the response to immune checkpoint inhibitors (ICIs) and can also affect patient survival (45–48). We further analyzed the expression of immune checkpoint-related genes in patients with different DDR groups. Compared with the high DDR group, most of the immune checkpoint-related genes, such as *IDO1*, *CD244*, and *LAGLS9*, were highly expressed in the low DDR group, suggesting that patients in the low DDR group may be more sensitive to ICIs treatment. Patients with cancer who have mutations in the DDR genes can improve clinical outcomes after receiving ICIs treatment (49, 50). TMB reflects the quantity of mutations in cancer. These mutations are processed into neo-antigens and presented by major histocompatibility complex (MHC) proteins to T-cells (51). Higher TMB results in more neo-antigens, increasing the chances of T-cell recognition, and clinically correlates with better ICI outcomes (52). In our results, the TMB in the low DDR group was higher than that in the high DDR group, and the expression of mutated genes in tumor cells can generate neoantigens, indicating that the ability of the low DDR group to produce neoantigens is stronger, and the probability of generating neoantigens is higher, predicting that the immune therapy effect is better in the low DDR group.

Great progress has been made in the research of targeted DDR anti-tumor drugs in cancer treatment. Clinically, various selective and effective DDR inhibitors have emerged, including PARP inhibitors, DNA damage kinases ATR, CHK1, WEE1, and ATM inhibitors (53). These targeted DDR drugs have been studied in multiple tumors such as ovarian cancer, breast cancer, pancreatic cancer, and prostate cancer. For example, drugs such as Olaparib and Niraparib have brought significant changes to the treatment of breast cancer and ovarian cancer. However, the study of targeted DDR drugs in CC has just begun (54, 55). We used the IC50 drug sensitivity analysis method to analyze the drugs that may benefit CC patients with high/low DDR status. Our study suggests that patients in the high DDR group may benefit from DNA damage repair-related drugs such as BI.2536, Bleomycin, Camptothecin, Cytarabine, and Doxorubicin, while patients in the low DDR group may benefit from the treatment of Veliparib, AKT inhibitor VIII, and CGP.60474. BI.2536 is a Plk1 enzyme inhibitor that is believed to induce mitotic arrest and a subsequent surge in apoptosis (56). Studies have shown that BI.2536 sensitizes oesophageal squamous cell carcinoma cells to cisplatin by inhibiting DDR pathways and inducing pyroptosis (57), and similar findings were also found in gastric cancer (58). But the application of BI.2536 in CC has not yet been discovered. Therefore, we speculate that it may become a potential new drug for the treatment of refractory CC. Our drug sensitivity analysis results can provide a basis for selecting targeted DDR drugs for CC patients and provide new ideas for developing new DDR-related targeted therapeutic drugs.

Through integrating second-generation sequencing and single-cell sequencing technologies, we conducted multi-omics analysis and constructed a DDR prognostic signature, which can serve as an independent prognostic indicator for CC patients. In addition, this signature can also reflect the tumor’s mutation and immune status, which is closely related to the development of cancer, and will contribute to the personalized treatment of CC patients. However, our study has certain limitations, and the clinical utility of key genes in the signature needs to be further validated in prospective trials. In addition, biological experiments are needed to elucidate the biological mechanisms between key genes in the signature and cancer markers such as DDR and immune response.

## 5 Conclusion

We constructed a DDR-related gene prognostic signature in CC. Using this signature, we can effectively evaluate the prognosis and immune microenvironment of CC patients, and provide references for patient treatment. We also demonstrated through cell experiments the role of *ITGB1* in CC and its influence on DDR, which provides a potential therapeutic target for CC.

## Data availability statement

The datasets presented in this study can be found in online repositories. The names of the repository/repositories and accession number(s) can be found in the article/**Supplementary Material**.

## Ethics statement

The studies involving human participants were reviewed and approved by Ethics Committee of Hunan Cancer Hospital. The patients/participants provided their written informed consent to participate in this study.

## Author contributions

YoZ, LL, and XP designed the study. JJ collected the data. YaZ for scripted debugging. XX and JK performed the data analysis and interpreted the data. XX and JK drafted the manuscript. YoZ and LL revised the manuscript. XX, JK, and JJ completed the cell experiment. All authors contributed to the article and approved the submitted version.
