# Supplementary material for: A novel DNA damage repair-related gene signature predicting survival, immune infiltration and drug sensitivity in cervical cancer based on single cell sequencing
Source: Front Immunol. 2023 Jun 28;14:1198391. doi: 10.3389/fimmu.2023.1198391 (PMC10337997; doi:10.3389/fimmu.2023.1198391)
Supplement: Supplementary file 1 [file DataSheet_1.pdf]

Supplementary Table S1 | shRNA sequences

ID	5’	stem	loop	stem	3’
ITGB1-RNAi(16737-1)-a	Ccgg	CCTGTTTACAAGGAGCTGAAA	CTCGAG	TTTCAGCTCCTTGTAAACAGG	TTTTTg
ITGB1-RNAi(16737-1)-b	aattcaaaaa	CCTGTTTACAAGGAGCTGAAA	CTCGAG	TTTCAGCTCCTTGTAAACAGG	
ITGB1-RNAi(16738-1)-a	Ccgg	GCCTTGCATTACTGCTGATAT	CTCGAG	ATATCAGCAGTAATGCAAGGC	TTTTTg
ITGB1-RNAi(16738-1)-b	aattcaaaaa	GCCTTGCATTACTGCTGATAT	CTCGAG	ATATCAGCAGTAATGCAAGGC	
ITGB1-RNAi(16739-1)-a	Ccgg	CCAAATCATGTGGAGAATGTA	CTCGAG	ACATTCTCCACATGATTTGG	TTTTTg
ITGB1-RNAi(16739-1)-a	aattcaaaaa	CCAAATCATGTGGAGAATGTA	CTCGAG	ACATTCTCCACATGATTTGG	

Supplementary Table S2 | Primer sequences

GAPDH	FORWARD	CTGCCAACGTGTCAGTGGTG
	REVERSE	TCAGTGTAGCCCAGGATGCC
ITGB1	FORWARD	TGGGCTTTACGGAGGAAGTAGAGG
	REVERSE	GACACTTGGGACTTTCAGGGATGC
ZC3H13	FORWARD	CAGAGGTGACAGAAGCAGAGCATAC
	REVERSE	GCAGCAGTAGTGGCAGCAAGAG
TOMM20	FORWARD	CAGAGGTGACAGAAGCAGAGCATAC
	REVERSE	GCAGCAGTAGTGGCAGCAAGAG

Supplementary Table S3| Annotation of cell surface annotator genes

gene	Cell Type
CD28A	Lymphocytos
CD27	Lymphocytos
PRF1	Lymphocytos
CD163	macrophages
FCGR2A	macrophages
COL1A2	Fibroblasts
APOD	Fibroblasts
PECAM1	Endothelial cells
EGFL7	Endothelial cells
EMCN	Endothelial cells

ACTG2	smooth muscle cells
EPCAM	Tumor/epithelial cells
CDH1	Tumor/epithelial cells
CDKN2A	Tumor/epithelial cells

Supplementary Table S4 | WGCNA and single-cell sequencing data analysis of intersecting genes

1	RPS3A	192	PSMD2
2	RPL34	193	MRGPRF
3	RPL32	194	ATP11B
4	RPL3	195	ISLR
5	RPS14	196	TCEAL7
6	MGP	197	TMOD3
7	RPL14	198	SPOCK1
8	RPS24	199	COL5A2
9	RPS8	200	MAT2A
10	RPS13	201	HAND2
11	RPL13	202	FBXL7
12	RPL15	203	RCC2
13	RPL5	204	PSMC6
14	RPL7	205	SDC2
15	RPL29	206	DLG1
16	RPL23A	207	DNAJB11
17	RPS27A	208	BAZ2B
18	RPL30	209	PKD2
19	RPL23	210	DCHS1
20	RPL12	211	NAA50
21	RPL10A	212	PURA
22	RPL6	213	PODN
23	TIMP2	214	ZFYVE16
24	RPL38	215	FKBP3
25	RPL13A	216	DENND1B
26	DCN	217	AFF1
27	THBS1	218	MACF1
28	SPARCL1	219	CACNA1C
29	RPS11	220	METAP1
30	RPLP1	221	AFF4
31	RPS3	222	TJP2
32	RARRES2	223	TMED10
33	RPL11	224	NOVA1
34	FGF7	225	MED13L
35	RPLP2	226	ACTR10
36	COL3A1	227	COL15A1

37	CALD1	228	PKD1
38	TAGLN	229	PRKG1
39	RPL7A	230	LYPLA1
40	RPS20	231	MEF2C
41	COL1A1	232	JMY
42	SPARC	233	GPRASP1
43	RPS9	234	FMOD
44	RPS6	235	SERINC1
45	RPS21	236	PAPSS1
46	RPSA	237	ZNF37A
47	PRRX1	238	ITGB1
48	SFRP4	239	EPDR1
49	COL6A2	240	PDE1A
50	RPL8	241	RSBN1
51	EIF3E	242	PLEKHF2
52	RPL19	243	SON
53	RPS18	244	RAB11FIP1
54	RPS15A	245	EIF4G1
55	RPL18A	246	ADAMTS5
56	FN1	247	TGFB3
57	COL6A1	248	MSRB3
58	AEBP1	249	CPOX
59	RPL27A	250	ITGA11
60	MYL9	251	FNIP1
61	RPS16	252	AVPR1A
62	RPL18	253	CDKN2AIP
63	COL6A3	254	FBN1
64	SERPINF1	255	ASPN
65	RPLP0	256	BOD1L1
66	RPS5	257	CACNA1H
67	GEM	258	TNPO1
68	COLEC12	259	SRRM2
69	NACA	260	RPL28
70	CXCL12	261	KTN1
71	MYH11	262	NCOA3
72	ZEB1	263	RPS19
73	RPL36	264	MRPS22
74	UBA52	265	FAM199X
75	HNRNPH1	266	ATP2B4
76	DDX5	267	UHMK1
77	MYLK	268	EBF1
78	COL1A2	269	RAB11FIP2
79	FZD6	270	FRZB

80	RPL31	271	PCMTD2
81	BNC2	272	CD46
82	REV3L	273	MAPK6
83	RPL27	274	HIPK3
84	BGN	275	NUMA1
85	FAM53C	276	TOMM20
86	CRISPLD2	277	PAIP2
87	EMILIN1	278	PRKCI
88	PGR	279	CHD1
89	MMP2	280	ASH1L
90	DES	281	PHIP
91	CNN1	282	HBP1
92	CCNI	283	RCOR1
93	LUM	284	PCF11
94	LEFTY2	285	ECHDC1
95	LMOD1	286	AKT1
96	COL12A1	287	MTDH
97	RPS2	288	APH1A
98	COL5A1	289	PNISR
99	OGN	290	LDB2
100	PCP4	291	RB1CC1
101	EIF3L	292	DDX17
102	GSKIP	293	REL
103	GREM1	294	ZNF106
104	CREBRF	295	SLC25A44
105	DDR2	296	MDM4
106	PCOLCE	297	RBBP6
107	MXRA8	298	CFAP97
108	RPL4	299	PTPN13
109	ACTA2	300	ZMYND11
110	RAB10	301	ANK2
111	PRELP	302	ABI1
112	RERE	303	STK24
113	THBS2	304	DLC1
114	EMX2	305	MORF4L1
115	MFAP4	306	ZNF280D
116	ZCCHC24	307	PGM2
117	NDN	308	CDC42BPB
118	NFATC4	309	KIF1B
119	GNG11	310	NCOA4
120	EIF4A2	311	ANKRD12
121	BCLAF1	312	UBE2V2
122	EIF3F	313	ACTL6A

123	ESRP1	314	NUMB
124	PTGER2	315	AKAP9
125	MASP1	316	TNRC6A
126	RPS7	317	SETD7
127	CTSK	318	ZMIZ1
128	EFEMP2	319	ZEB2
129	PDGFRB	320	PLN
130	ACTG2	321	VPS4B
131	A2M	322	VTI1B
132	PJA2	323	EIF4E
133	LAMA4	324	PRKAR1A
134	POLG2	325	RPS29
135	EIF3H	326	EIF2S1
136	PMP22	327	ADRA2A
137	PDZRN3	328	ACAP2
138	SMC4	329	ZNF292
139	COL14A1	330	PRRC2B
140	ATG3	331	KMT2A
141	RUNX1T1	332	RAB7A
142	ITGA8	333	GPBP1
143	ITPR3	334	BAZ1A
144	SYNE1	335	SEC62
145	FILIP1L	336	SEC63
146	MEIS3	337	CD164
147	ANTXR1	338	SNX13
148	SOD3	339	AMD1
149	TMX1	340	RIF1
150	TNRC6B	341	DDX24
151	IMPDH2	342	CMIP
152	PEG3	343	RSRC2
153	AP2M1	344	RAP1B
154	CPXM1	345	ROCK2
155	PSMC1	346	SPCS3
156	TCEAL2	347	MYO6
157	ATP2C1	348	HNRNPF
158	COL4A2	349	ARIH1
159	SYNE2	350	WAC
160	JMJD1C	351	CLDND1
161	CNRIP1	352	SPIN1
162	RPL35	353	CNOT2
163	EDNRA	354	COPB2
164	FGFR1	355	ARHGAP21
165	FOXO3	356	DICER1

166	PHF3	357	NFAT5
167	FAP	358	ADAR
168	TPM3	359	FXR1
169	ADAMTS9	360	MARK3
170	MBNL1	361	DYNC1H1
171	CDCA4	362	BPTF
172	MYOCD	363	YME1L1
173	MCM4	364	CLEC11A
174	SMAD5	365	PCMTD1
175	PDCD10	366	CNIH1
176	DDX3X	367	SNX6
177	NUCKS1	368	HNRNPDL
178	YWHAZ	369	RGS5
179	FAM133B	370	RSF1
180	SORBS1	371	AQP1
181	HNRNPA0	372	TCP1
182	PPP1R12A	373	SRSF11
183	ATRX	374	C1orf43
184	B3GNT5	375	NUFIP2
185	CDH11	376	PTK2
186	RAB23	377	TRA2B
187	ZC3H13	378	SRP9
188	AHI1	379	PAPOLA
189	ABL1	380	ZNF706
190	PSMD7	381	TUBA1B
191	COL4A1		

Supplementary Table S5 | Univariate COX analysis to obtain genes associated with patient prognosis

ID	Hazard_ratio	Low_CI	Ligh_CI	p-value
CD46	1.010464	1.00656	1.014384	1.37E-07
ITGB1	1.008379	1.003697	1.013082	0.000442
TCP1	1.00739	1.002651	1.012151	0.002209
TOMM20	1.005092	1.001806	1.008389	0.002367
EFEMP2	1.016719	1.005386	1.028181	0.003743
TPM3	1.010388	1.003197	1.01763	0.004569
ZC3H13	1.031757	1.008761	1.055277	0.006559
ROCK2	1.028905	1.006893	1.051397	0.009806
AMD1	1.022631	1.005341	1.040218	0.010103
LAMA4	1.027779	1.00606	1.049967	0.011925
MFAP4	1.001878	1.000383	1.003375	0.013796
C1orf43	1.003156	1.000626	1.005692	0.014474
ZCCHC24	1.031625	1.005519	1.05841	0.017276

NFATC4	1.022707	1.003891	1.041876	0.017796
ADAMTS9	1.014264	1.002189	1.026484	0.020457
JMJD1C	1.026849	1.004039	1.050178	0.020797
SERINC1	1.00439	1.000623	1.008171	0.022334
RAP1B	1.059488	1.007413	1.114255	0.024628
TMOD3	1.015313	1.001896	1.02891	0.02515
CNIH1	1.012901	1.001432	1.024502	0.02737
ITPR3	1.006691	1.0007	1.012716	0.028529
SEC63	1.017152	1.001677	1.032866	0.029696
TJP2	1.994148	1.065401	3.732515	0.030925
REV3L	1.042102	1.003674	1.082	0.031453
TMED10	1.002749	1.000242	1.005262	0.031569
MTDH	1.010638	1.000734	1.020641	0.035206
COL3A1	1.000216	1.000015	1.000418	0.035655
ABL1	1.011449	1.00075	1.022263	0.0359
MMP2	1.001164	1.00006	1.00227	0.03876
ATG3	0.983468	0.967461	0.999739	0.046469
EIF3E	1.019454	1.000238	1.039039	0.047208
MSRB3	1.021811	1.000203	1.043887	0.047863
EIF3H	1.004709	1.000007	1.009432	0.049652
