# Supplementary material for: A novel DNA damage repair-related gene signature predicting survival, immune infiltration and drug sensitivity in cervical cancer based on single cell sequencing
Source: Front Immunol. 2023 Jun 28;14:1198391. doi: 10.3389/fimmu.2023.1198391 (PMC10337997; doi:10.3389/fimmu.2023.1198391)
Supplement: Supplementary file 2 [file DataSheet_2.pdf]

High DDRscore (n = 128) v/s Low DDRscore (n = 13ligh DDRscor.ow DDRscor OR P−value

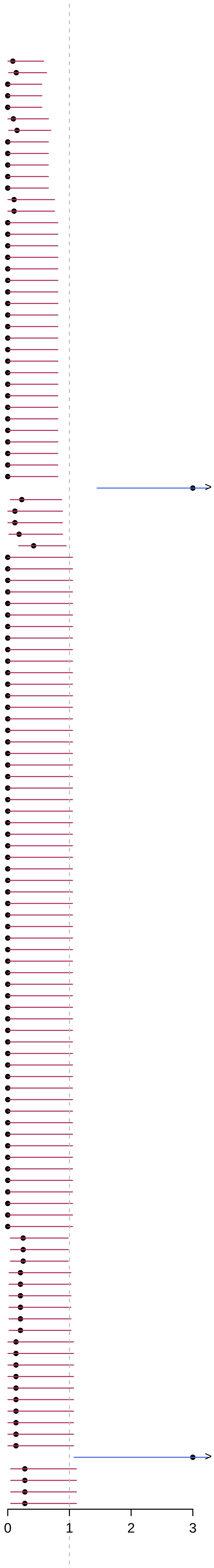

			OR	P−value
KIAA1109	11	1	0.082	**
TCHH	13	2	0.138	**
KIF1A	8	0	0	**
PIK3R4	8	0	0	**
TTC28	8	0	0	**
ARHGAP5	10	1	0.091	**
ALMS1	12	2	0.151	**
DMBT1	7	0	0	**
DMWD	7	0	0	**
FBN3	7	0	0	**
STARD9	7	0	0	**
TLL2	7	0	0	**
CNTRL	9	1	0.102	**
HECTD4	9	1	0.102	**
ASCC3	6	0	0	*
CD1A	6	0	0	*
CFI	6	0	0	*
DOCK5	6	0	0	*
FASN	6	0	0	*
GABRG3	6	0	0	*
IL16	6	0	0	*
KDM4B	6	0	0	*
KIF6	6	0	0	*
KLHL11	6	0	0	*
MED17	6	0	0	*
MLH3	6	0	0	*
NUP62	6	0	0	*
PRAMEF4	6	0	0	*
PWWP2A	6	0	0	*
SH3PXD2B	6	0	0	*
SLK	6	0	0	*
SMARCA2	6	0	0	*
THAP9	6	0	0	*
TRERF1	6	0	0	*
TTLL7	6	0	0	*
USP24	6	0	0	*
ZNF354C	6	0	0	*
FBXL20	0	7	Inf	*
NWD1	12	3	0.228	*
CASKIN2	8	1	0.116	*
MST1R	8	1	0.116	*
CIC	10	2	0.184	*
SYNE1	23	11	0.42	*
A2ML1	5	0	0	*
ACAD10	5	0	0	*
ADNP	5	0	0	*
ALOX5	5	0	0	*
ALPK1	5	0	0	*
APLP1	5	0	0	*
ARID4B	5	0	0	*
ATP8B1	5	0	0	*
ATXN2L	5	0	0	*
BMPR1A	5	0	0	*
BRE	5	0	0	*
C20orf194	5	0	0	*
CCDC150	5	0	0	*
CDK11B	5	0	0	*
CEP164	5	0	0	*
CHAD	5	0	0	*
CNTN4	5	0	0	*
CPQ	5	0	0	*
CROT	5	0	0	*
DAZAP1	5	0	0	*
DDB1	5	0	0	*
GJD2	5	0	0	*
HAUS6	5	0	0	*
IQSEC1	5	0	0	*
KCNJ12	5	0	0	*
KLHL2	5	0	0	*
LRRC4B	5	0	0	*
MAATS1	5	0	0	*
MAP3K9	5	0	0	*
MAP7D1	5	0	0	*
NAT10	5	0	0	*
OR8U1	5	0	0	*
PAXBP1	5	0	0	*
PCYOX1L	5	0	0	*
PIK3R3	5	0	0	*
PLEKHG1	5	0	0	*
PNN	5	0	0	*
RGPD3	5	0	0	*
SARDH	5	0	0	*
SLC12A5	5	0	0	*
SLC26A3	5	0	0	*
SMC2	5	0	0	*
SRRM4	5	0	0	*
SYMPK	5	0	0	*
SYNJ1	5	0	0	*
TARBP1	5	0	0	*
TELO2	5	0	0	*
TGM4	5	0	0	*
TMCO6	5	0	0	*
TPST2	5	0	0	*
TRAPPC12	5	0	0	*
TXNRD1	5	0	0	*
UPF3B	5	0	0	*
USP28	5	0	0	*
VPS52	5	0	0	*
VWA3B	5	0	0	*
WAC	5	0	0	*
ZNF551	5	0	0	*
ZNF676	5	0	0	*
DNAH7	11	3	0.25	*
FBN2	11	3	0.25	*
NBEAL1	11	3	0.25	*
FLT1	9	2	0.206	*
MDC1	9	2	0.206	*
STK11	9	2	0.206	*
TCOF1	9	2	0.206	*
TDRD6	9	2	0.206	*
UTRN	9	2	0.206	*
ARPP21	7	1	0.134	*
KIAA0430	7	1	0.134	*
KIAA1549	7	1	0.134	*
MYH14	7	1	0.134	*
NOD2	7	1	0.134	*
PCDHB2	7	1	0.134	*
PHLPP1	7	1	0.134	*
RP1L1	7	1	0.134	*
SMCHD1	7	1	0.134	*
TRPC1	7	1	0.134	*
SON	1	8	8.209	*
BRCA2	10	3	0.278	*
FBN1	10	3	0.278	*
MACF1	10	3	0.278	*
MAP1B	10	3	0.278	*

Odds ratio with 95% CI
(1 = no effect, < 1 High DDRscore has more mutants)
