# Supplementary figures and images for: A novel DNA damage repair-related gene signature predicting survival, immune infiltration and drug sensitivity in cervical cancer based on single cell sequencing
Source: Front Immunol. 2023 Jun 28;14:1198391. doi: 10.3389/fimmu.2023.1198391 (PMC10337997; doi:10.3389/fimmu.2023.1198391)

Altered in 20 (7.72%) of 259 samples.

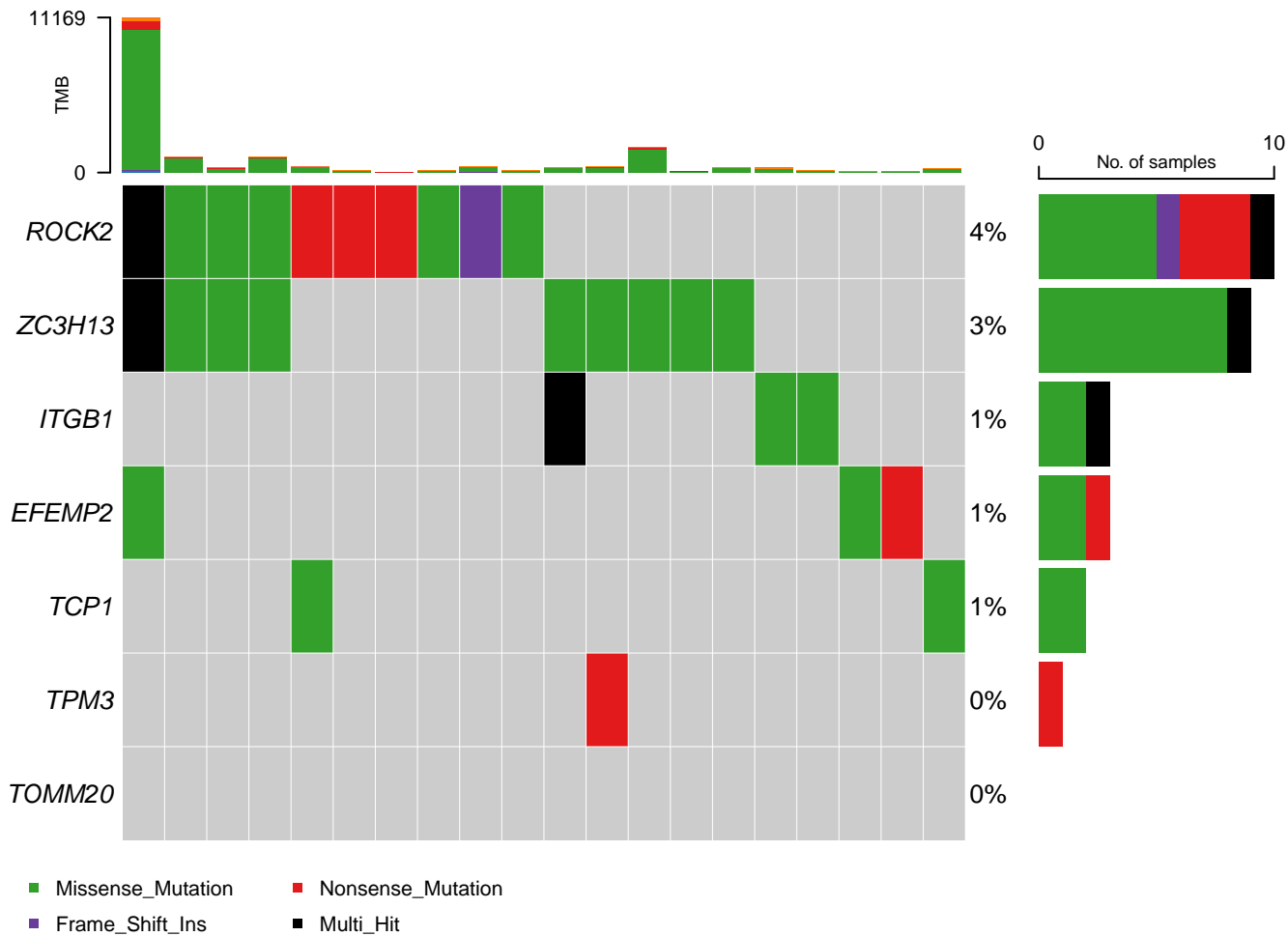

Supplement: Supplementary file 3 [file DataSheet_3.pdf]

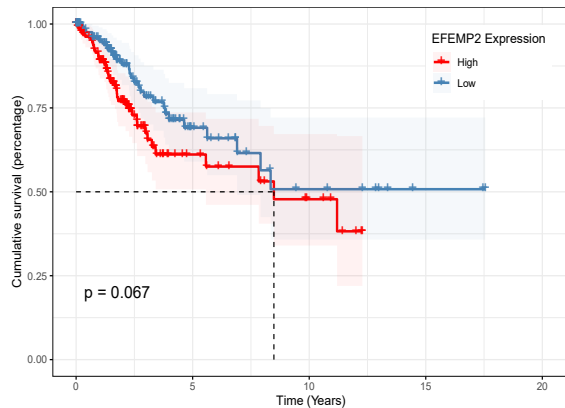

Number at risk

|      |     |    |   |   |   |
|------|-----|----|---|---|---|
| High | 141 | 18 | 7 | 0 | 0 |
| Low  | 142 | 24 | 8 | 2 | 0 |

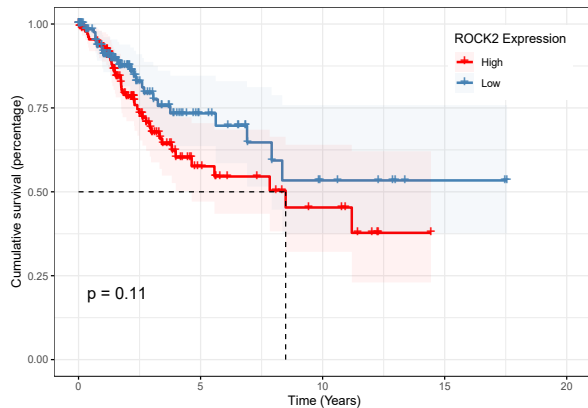

Number at risk

|      |     |    |   |   |   |
|------|-----|----|---|---|---|
| High | 141 | 20 | 8 | 0 | 0 |
| Low  | 142 | 22 | 7 | 2 | 0 |

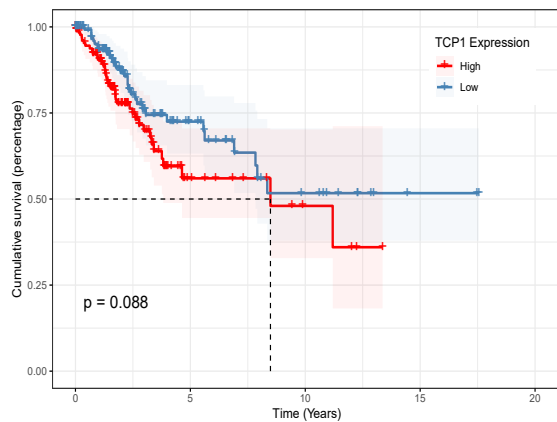

Number at risk

|      |     |    |    |   |   |
|------|-----|----|----|---|---|
| High | 141 | 13 | 4  | 0 | 0 |
| Low  | 142 | 29 | 11 | 2 | 0 |

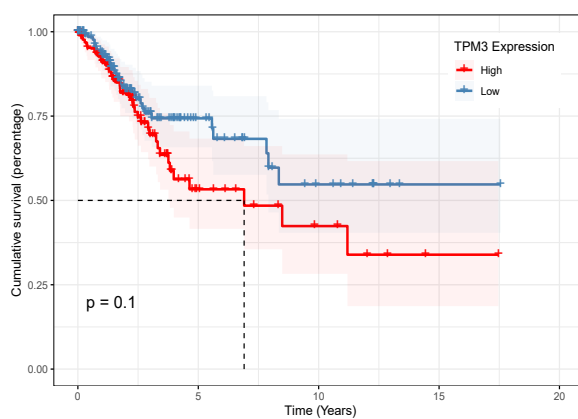

Number at risk

|      |     |    |   |   |   |
|------|-----|----|---|---|---|
| High | 141 | 15 | 6 | 1 | 0 |
| Low  | 142 | 27 | 9 | 1 | 0 |

Supplement: Supplementary file 4 [file DataSheet_4.pdf]
